# A Generalized Model for Curved Nanobeams Incorporating Surface Energy

**DOI:** 10.3390/mi14030663

**Published:** 2023-03-16

**Authors:** Mahmoud E. Khater

**Affiliations:** 1 Mechanical Engineering Department, College of Engineering and Physics, KFUPM, Dhahran 31261, Saudi Arabia; mkhater@kfupm.edu.sa; 2 Interdisciplinary Research Center of Hydrogen and Energy Storage, KFUPM, Dhahran 31261, Saudi Arabia

**Keywords:** curved nanobeams, Euler–Bernoulli beam model, surface energy

## Abstract

This work presents a comprehensive model for nanobeams, incorporating beam curvature and surface energy. Gurtin–Murdoch surface stress theory is used, in conjunction with Euler–Bernoulli beam theory, to model the beams and take surface energy effects into consideration. The model was validated by contrasting its outcomes with experimental data published in the literature on the static bending of fixed–fixed and fixed–free nanobeams. The outcomes demonstrated that surface stress alters the stiffness of both fixed–fixed and fixed–free nanobeams with different behaviors in each case.

## 1. Introduction

Nanotechnology is a field of science that focuses on the design and implementation of materials and devices on the nanoscale. It has the potential to revolutionize a wide range of industries, from electronics and medicine to energy and environmental sustainability. In recent years, there have been several notable advances in nanotechnology, especially in nanomaterials and nano-electronics [1,2,3].

Nanomaterials exhibit distinctive optical, electrical, and mechanical characteristics, which have drawn the attention of the scientific community as well as of industry. Surface energy effects are provoked by the high surface to volume ratio of nanostructures [4]. Surface energy results from the difference in bonding forces between atoms in the bulk of the material and those in the surface layer at extremely small scales. As a result, mechanical and electrical properties of the material may alter. The elastic modulus of the material could increase by up to three times as a result of surface effects, which encompass surface stress, surface roughness, and surface oxidation [5].

The relationship between material size and its mechanical characteristics, including its Young’s modulus and flexural rigidity, has been experimentally studied. Surfaces effects were examined by He and Lilley [6,7] for nanowires at various boundary conditions in order to determine their effects on elasticity and resonant frequencies in bending. Wong et al. [8] statically measured the modulus of elasticity for nanorods made of silicon carbide using an AFM probe. On the other hand, Poncharal et al. [9] measured the modulus of elasticity dynamically by resonantly exciting carbon nanotubes. Jing et al. [5] experimentally proved that surface energy results in decreasing Young’s modulus for silver nanowires of diameters above 100 nm.

Since they do not account for the impacts of surface stresses, classic continuum models fall short in providing accurate predictions for the behavior of nanomaterials [10,11]. Gurtin and Murdoch [12] developed a continuum model incorporating surface energy for isotropic materials. In this approach, the surface layer is modeled as a membrane that perfectly adheres to the bulk material.

Based on Gurtin–Murdoch theory, Lu et al. [10] devised a model for nanoplates, including surface effects. Their work is an extension of the work of Lim and He [13] on thin films. Huang [14] applied Gurtin–Murdoch formalism to simply-supported thin films to study their buckling, bending, and free vibration. Liu and Rajapakse [11] devised a model for nanobeams based on Gurtin–Murdoch theory. They derived analytic solutions for static bending of nanobeams, including surface stresses for various boundary conditions, implementing Euler and Timoshenko beam formulations. They also found formulae for their mode shapes under free vibration. They extended their work to study nanobeam bending, buckling, and free vibration using finite elements [15] and the energy approach [16]. Feng et al. [17] and Xia et al. [18] used the Gurtin–Murdoch model to predict the effective modulus of elasticity for nanoporous materials.

The characterization of nanostructures should take curvature into account, since carbon nanotubes exhibit substantial waviness and curvature throughout their length [19]. This means that, in addition to the impacts of surface stresses, it is also essential to take into consideration the beam curvature when modeling nanobeams. Beam curvature was investigated by several researchers. Based on the classic Euler’s elastica theory for beams with large deflection, Liu et al. [20] presented an analytical model to study large deflection of a curved nanobeam with rectangular cross-section. They considered a surface stress tensor, based on the general Young–Laplace equation [21]. Other researchers modeled curved nanobeams modeling the beams as functionally-graded materials [22], while others modeled them using the non-local elasticity theory [23].

It has been shown that it is necessary to take into consideration the impacts of beam curvature and surface energy in modeling nanobeams. Devising a model that considers these elements was the objective of this study. The model is founded on the Euler–Bernoulli beam theory, taking initial beam curvature into account. Further, the Gurtin–Murdoch surface elasticity theory is utilized to account for surface energy effects. Two case studies of fixed–fixed and fixed–free nanobeams are given to examine the response of such beams under static loading.

## 2. Model

**Figure 1:**
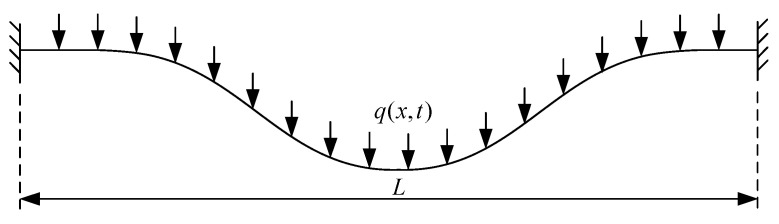
Fixed-fixed beam.

**Figure 2:**
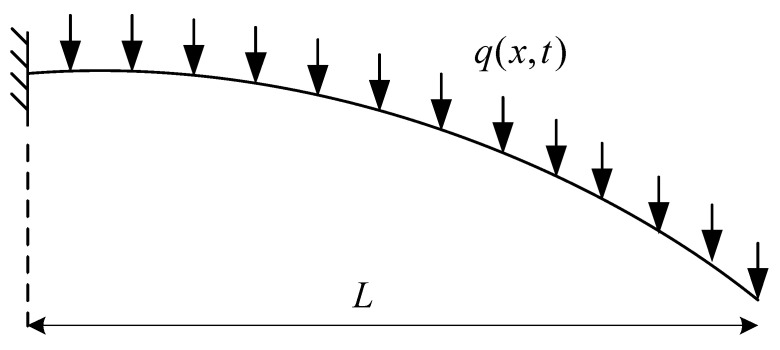
Fixed-free beam.

Consider nanobeams of fixed–fixed, Figure 1, and fixed–free, Figure 2, boundary conditions with initial curvature and length *L*. A distributed force of intensity q(x) is assumed to apply over the beam span.

**Figure 3:**
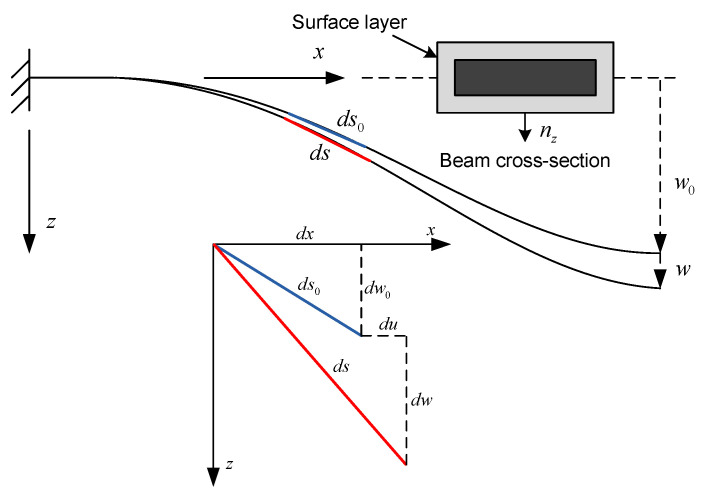
A segment of the nanobeam showing deflection *w* beyond initial curvature height $w_{0}$. An element along beam span is shown elongating from $ds_{0}$ to ds due to beam deflection.

Figure 3 shows a segment of a curved beam before and after elongation due to curvature. Displacement in the axial direction is denoted by *u* and displacement in the transverse direction is denoted by *w*.

For a beam with initial curvature, the length of an infinitesimal element is given by

(1) $$\begin{array}{cc} ds_{o} & =\sqrt{dx^{2}+dw_{o}^{2}}\\ \frac{ds_{o}}{dx} & =\sqrt{1+w_{o}^{\prime 2}} \end{array}$$

where primes denote derivatives with respect to *x*. After elongation,

(2) $$\begin{array}{cc} ds & =\sqrt{{\left(dx+du\right)}^{2}+{\left(dw_{o}+dw\right)}^{2}}\\ \frac{ds}{dx} & =\sqrt{{\left(1+u^{\prime }\right)}^{2}+{\left(w_{o}^{\prime }+w^{\prime }\right)}^{2}} \end{array}$$

The strain due to mid-plane elongation is expressed as

(3) $$\begin{array}{cc} \epsilon_{o} & =\frac{ds-ds_{o}}{ds_{o}}\\ & =\frac{ds}{ds_{o}}-1=\frac{ds/dx}{ds_{o}/dx}-1\\ & =\frac{\sqrt{{\left(1+u^{\prime }\right)}^{2}+{\left(w_{o}^{\prime }+w^{\prime }\right)}^{2}}}{\sqrt{1+w_{o}^{\prime 2}}}-1\\ \; & \approx u^{\prime }+w^{\prime }w_{0}^{\prime }+\frac{1}{2}w^{\prime 2}+\cdots \end{array}$$

after expanding in a Taylor series and neglecting infinitesimally small terms. Adding the effect of flexural strain according to the Euler–Bernoulli beam theory, the total strain is given by

(4) $$\begin{array}{c} \epsilon_{x}=u^{\prime }+w^{\prime }w_{0}^{\prime }+\frac{1}{2}w^{\prime 2}-z\left(w^{\prime \prime }+w_{0}^{\prime \prime }\right) \end{array}$$

According to the Gurtin–Murdoch model, a surface layer is assumed to exist and perfectly adhere to the bulk material. Assuming a plane–stress state, traction forces between the material bulk and the surface layer are represented by an axial component $T_{x}$ and a transverse component $T_{z}$. The bulk and surface stresses are expressed as [10,11,12]

(5) $$\begin{array}{c} \tau_{xx,x}-T_{x}=\rho_{s}\;{\ddot{u}}^{s} \end{array}$$

(6) $$\begin{array}{c} \tau_{zx,x}-T_{z}=\rho_{s}\;{\ddot{w}}^{s} \end{array}$$

where $\tau_{ij,k}=\partial \tau_{ij}/\partial k$ and $\rho_{s}$ is surface layer density.

Assuming that the bulk and surface materials are isotropic and linearly elastic, the constitutive relations are expressed as [10,11,12]

(7) $$\begin{array}{cc} \tau_{xx} & =\tau_{s}+\left(2\mu_{s}+\lambda_{s}\right)\;\epsilon_{x} \end{array}$$

(8) $$\begin{array}{cc} \tau_{zx} & =\tau_{s}\;\left(w^{\prime }+w_{o}^{\prime }\right) \end{array}$$

where $\tau_{s}$ is the surface stress and $\tau_{s},\lambda_{s}$ are surface Lamé constants.

Assuming that the bending stress distribution varies linearly across the beam cross-section [10,11], the bending stress at any point in the cross-section is given by

(9) $$\begin{array}{c} \sigma_{z}=\sigma_{0}+\frac{z}{h}\left(\sigma_{z}^{t}-\sigma_{z}^{b}\right) \end{array}$$

where the distance between top and bottom surface layers $h=w^{t}-w^{b}$, $\sigma_{z}^{t}$ and $\sigma_{z}^{b}$ are the bending stresses at the top and bottom surface layers, respectively. The bending stress at the beam center-line $\sigma_{0}$ is assumed to be the average value of $\sigma_{z}^{t}$ and $\sigma_{z}^{b}$ [10,11], i.e., $\sigma_{0}=\left(\sigma_{z}^{t}+\sigma_{z}^{b}\right)/2$.

Substituting with Equations (5) and (6) into Equation (9), noting that $T_{z}=\sigma_{z}$ at the surface and using Equation (8), yields

(10) $$\begin{array}{cc} \sigma_{z} & =\frac{1}{2}\left(\tau_{zx,x}^{t}-\rho_{s}\;{\ddot{w}}^{t}+\tau_{zx,x}^{b}-\rho_{0}\;{\ddot{w}}^{b}\right)\\ & +\frac{z}{h}\left(\tau_{zx,x}^{t}-\rho_{s}\;{\ddot{w}}^{t}-\tau_{zx,x}^{b}+\rho_{s}\;{\ddot{w}}^{b}\right)\\ & =\frac{1}{2}\left[\tau_{s}\left(w^{\prime \prime t}-w^{\prime \prime b}\right)-\rho_{s}\;\left({\ddot{w}}^{t}-{\ddot{w}}^{b}\right)\right]\\ \; & +\frac{z}{h}\left[\tau_{s}\left(w^{\prime \prime t}-w^{\prime \prime b}\right)-\rho_{s}\;\left({\ddot{w}}^{t}-{\ddot{w}}^{b}\right)\right] \end{array}$$

Using Equation (7), Equation (10) can be rewritten as

(11) $$\begin{array}{cc} \sigma_{z} & =\frac{1}{2}\left[\tau_{s}\left(w^{\prime \prime t}-w^{\prime \prime b}\right)-\rho_{s}\;\left({\ddot{w}}^{t}-{\ddot{w}}^{b}\right)\right]\\ \; & +\frac{z}{h}\left[\tau_{s}\left(w^{\prime \prime t}+w^{\prime \prime b}\right)-\rho_{s}\;\left({\ddot{w}}^{t}+{\ddot{w}}^{b}\right)\right] \end{array}$$

Assuming the same curvature and inertia on top and bottom surface layers, the first term on the right hand side of the above equation cancels out and the equation reduces to

(12) $$\begin{array}{c} \sigma_{z}=\frac{2z}{h}\left[\tau_{s}\left(w^{\prime \prime }+w_{0}^{\prime \prime }\right)-\rho_{s}\;\ddot{w}\right] \end{array}$$

where $w=(w^{t}+w^{b})/2$.

**Figure 4:**
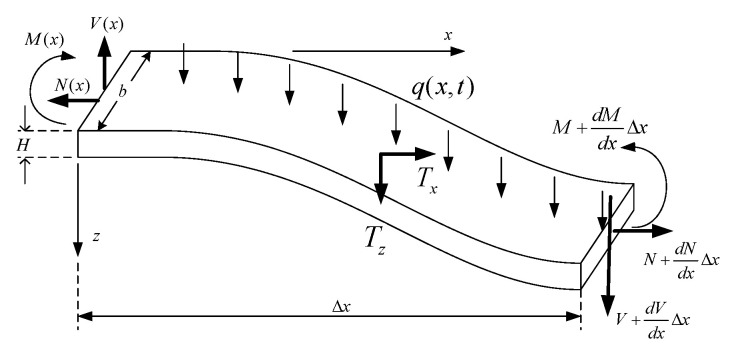
A beam element of rectangular cross-section.

**Figure 5:**
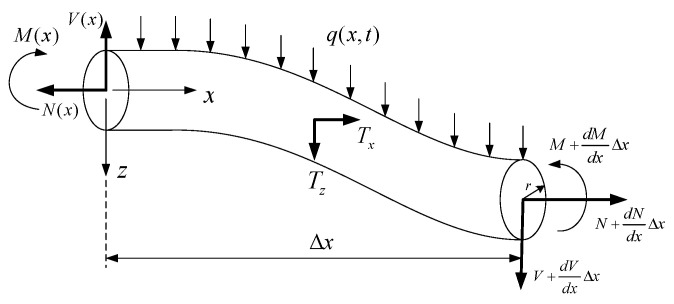
A beam element of circular cross-section.

Consider two beam elements of rectangular and circular cross-sections, as shown in Figure 4 and Figure 5, respectively. The cross-section in Figure 4 has width *b* and thickness *H* while that of Figure 5 has radius *r*, and both elements have length Δx and cross-sectional area *A*. On each beam element, *M* is the bending moment, *V* is the shearing force, and *N* is the axial force acting normal to the cross-section.

Summing forces in *z* direction,

$$\begin{array}{cc} & \Sigma F_{z}=m\;a_{z}\\ & V+\frac{dV}{dx}\;\Delta x-V+q\left(x,t\right)\;\Delta x+\Delta x\int T_{z}\;ds=\Delta x\int \rho \;\ddot{w}\;dA \end{array}$$

yielding

(13) $$\begin{array}{c} \frac{dV}{dx}+q\left(x,t\right)+\int T_{z}\;ds=\int \rho \;\ddot{w}\;dA \end{array}$$

where *s* denotes diplacement along the cross-section perimeter and the upper dots indicate derivatives with respect to time.

Taking moments about the fixed support,

$$\begin{array}{cc} \; & \Sigma M_{O}=I\;\ddot{\theta }\\ \; & M+\frac{dM}{dx}\;\Delta x-M+V\;\Delta x+\frac{dV}{dx}\;\Delta x\;\Delta x+\Delta x\int \left(\frac{h}{2}+z\right)T_{x}\;\Delta x\;ds\\ & +\frac{\Delta x}{2}\int T_{z}\;\Delta x\;ds+\frac{\Delta x}{2}\Delta x\;q\left(x,t\right)+\left(N+\frac{\partial N}{\partial x}\Delta x\right)\frac{\partial (w+w_{0})}{\partial x}\Delta x\\ \; & =-\;z\int \rho \;\ddot{u}\;\Delta x\;dA \end{array}$$

which reduces to

(14) $$\begin{array}{c} \frac{dM}{dx}+V+\int z\;T_{x}\;ds+N\;\frac{\partial (w+w_{0})}{\partial x}=0 \end{array}$$

neglecting rotary inertia and infinitesimally small higher-order terms.

Substituting with Equations (5) and (6) into Equations (13) and (14) yields

(15) $$\begin{array}{c} \frac{dV}{dx}+q\left(x,t\right)+\int \tau_{zx,x}\;ds-\int \rho_{s}\;{\ddot{w}}^{s}\;ds=\int \rho \;\ddot{w}\;dA \end{array}$$

(16) $$\begin{array}{c} \frac{dM}{dx}+V+N\frac{\partial w}{\partial x}-\int \tau_{xx,x}\;z\;ds+\int \rho_{s}\;{\ddot{u}}^{s}\;z\;ds=0 \end{array}$$

Taking the derivative of Equation (16), with respect to *x*, and substituting with Equation (15), neglecting surface inertia in *x*-direction, yields

(17) $$\begin{array}{cc} & \frac{d^{2}M}{dx^{2}}+N\frac{\partial^{2}\left(w+w_{0}\right)}{\partial x^{2}}-\frac{\partial }{\partial x}\int \tau_{xx,x}\;z\;ds-\int \tau_{zx,x}\;ds\\ \; & +\int \rho_{s}\;\ddot{w}\;ds+\int \rho \;\ddot{w}\;dA=q\left(x,t\right) \end{array}$$

The integral

(18) $$\begin{array}{cc} \int \tau_{xx,x}\;z\;ds & =\frac{\partial }{\partial x^{2}}\int \left[\tau_{s}\left(2\mu_{s}+\lambda_{s}\right)\left(u^{\prime }+w^{\prime }w_{0}^{\prime }+\frac{1}{2}w^{\prime 2}-z\left(w^{\prime \prime }+w_{0}^{\prime \prime }\right)\right)\right]z\;ds\\ & =\left(\frac{\partial \tau_{s}}{\partial x^{2}}+\left(2\mu_{s}+\lambda_{s}\right)\left[u^{\prime \prime \prime }+\frac{\partial }{\partial x^{2}}\left(w^{\prime }w_{o}^{\prime }+\frac{1}{2}w^{\prime 2}\right)\right]\right)\int z\;ds\\ & -\left(2\mu_{s}+\lambda_{s}\right)\left(\frac{\partial^{4}w}{\partial x^{4}}+\frac{d^{4}w_{o}}{dx^{4}}\right)\int z^{2}\;ds\\ \; & =-\left(2\mu_{s}+\lambda_{s}\right)I_{s}\left(\frac{\partial^{4}w}{\partial x^{4}}+\frac{d^{4}w_{o}}{dx^{4}}\right) \end{array}$$

where ∫zds=0, and $I_{s}=\int z^{2}\;ds$ for the surface.

The integral

(19) $$\begin{array}{c} \frac{\partial }{\partial x}\int \tau_{zx,x}\;ds=\frac{\partial }{\partial x}\int \tau_{s}\left(\frac{\partial^{2}w}{\partial x^{2}}+\frac{d^{2}w_{o}}{dx^{2}}\right)ds=\tau_{s}\;S_{s}\left(\frac{\partial^{2}w}{\partial x^{2}}+\frac{d^{2}w_{o}}{dx^{2}}\right) \end{array}$$

and $S_{s}=\int n_{z}\;ds$ where $n_{z}$ is a unit vector in *z*-direction.

The axial force on the beam cross-section is given by

(20) $$\begin{array}{cc} \tilde{N} & =\int (E\epsilon_{x}+\nu \sigma_{z})\;dA\\ & =\int E\left(u^{\prime }+w^{\prime }w_{0}^{\prime }+\frac{1}{2}w^{\prime 2}-z\left(w^{\prime \prime }+w_{0}^{\prime \prime }\right)\right)dA\\ & -\int \frac{2\nu }{h}\left(z\;\tau_{s}\left(w^{\prime \prime }+w_{0}^{\prime \prime }\right)-\rho_{s}\;\ddot{w}\right)dA\\ \; & =E\;A\left(u^{\prime }+w^{\prime }w_{0}^{\prime }+\frac{1}{2}w^{\prime 2}\right) \end{array}$$

where the first moment of area integral ∫zdA vanishes and surface inertia is neglected. Integrating over the beam length, assuming an externally applied compressive force $P_{c}$ to act on the beam, the total axial force, per unit length, generated in the beam can be expressed as

(21) $$\begin{array}{cc} N & =P_{c}+\frac{1}{L}\int_{0}^{L}E\;A\left(u^{\prime }+w^{\prime }w_{0}^{\prime }+\frac{1}{2}w^{\prime 2}\right)\;dx\\ \; & =P_{c}+\frac{EA}{2L}\int_{0}^{L}\left(w^{\prime 2}+2w^{\prime }w_{0}^{\prime }\right)\;dx \end{array}$$

where the integral $\int_{0}^{L}u^{\prime }dx$ vanishes at the boundaries for fixed–fixed beams, due to immobility, and for fixed–free beams, due to inextensibility [24].

The bending moment over the beam cross-section is given by

(22) $$\begin{array}{cc} M & =-\int z(E\epsilon_{x}+\nu \sigma_{z})\;dA\\ & =-\int Ez\left[u^{\prime }+w^{\prime }w_{0}^{\prime }+\frac{1}{2}w^{\prime 2}-z\left(w^{\prime \prime }+w_{0}^{\prime \prime }\right)\right]\;dA\\ & -\int \frac{2\nu }{h}z^{2}\left[\tau_{s}\left(w^{\prime \prime }+w_{0}^{\prime \prime }\right)-\rho_{s}\;\ddot{w}\right]dA\\ \; & =E\;I\left(w^{\prime \prime }+w_{0}^{\prime \prime }\right)-\frac{2\nu \;I}{h}\left[\tau_{s}\left(w^{\prime \prime }+w_{0}^{\prime \prime }\right)-\rho_{s}\;\ddot{w}\right] \end{array}$$

where $I=\int z^{2}dA$ is the second area moment of the beam cross-section.

Substituting with Equations (18), (19), (21) and (22) into Equation (17), adding linear viscous damping, yields

(23) $$\begin{array}{cc} \; & \int \rho \frac{\partial^{2}w}{\partial t^{2}}dA+\int \rho_{s}\frac{\partial^{2}w}{\partial t^{2}}ds+c\;\frac{\partial w}{\partial t}+\frac{2\nu \;I\;\rho_{s}}{h}\frac{\partial^{4}w}{\partial x^{2}\partial t^{2}}\\ \; & +\left(EI+\left(2\mu_{s}+\lambda_{s}\right)I_{s}-\frac{2\nu \;I\;\tau_{s}}{h}\right)\left(\frac{\partial^{4}w}{\partial x^{4}}+\frac{d^{4}w_{o}}{dx^{4}}\right)\\ \; & -\left(\frac{\partial^{2}w}{\partial x^{2}}+\frac{d^{2}w_{o}}{dx^{2}}\right)\left[P_{c}+\tau_{s}S_{s}+\frac{EA}{2L}\int_{0}^{L}\left(w^{\prime 2}+2w^{\prime }w_{0}^{\prime }\right)\;dx\right]=q\left(x,t\right) \end{array}$$

which can be further simplified to

(24) $$\begin{array}{cc} \; & \left(\rho \;A+\rho_{s}\;S_{s}\right)\frac{\partial^{2}w}{\partial t^{2}}+c\;\frac{\partial w}{\partial t}+\frac{2\nu \;I\;\rho_{s}}{h}\frac{\partial^{4}w}{\partial x^{2}\partial t^{2}}\\ \; & +\left(EI+\left(2\mu_{s}+\lambda_{s}\right)I_{s}-\frac{2\nu \;I\;\tau_{s}}{h}\right)\left(\frac{\partial^{4}w}{\partial x^{4}}+\frac{d^{4}w_{o}}{dx^{4}}\right)\\ \; & -\left(\frac{\partial^{2}w}{\partial x^{2}}+\frac{d^{2}w_{o}}{dx^{2}}\right)\left[P_{c}+\tau_{s}S_{s}+\frac{E\;A}{2L}\int_{0}^{L}\left(w^{\prime 2}+2w^{\prime }w_{0}^{\prime }\right)\;dx\right]=q\left(x,t\right) \end{array}$$

Equation (24) represents a generalized model for an Euler–Bernoulli curved nanobeam incorporating surface energy under compressive and transverse loading.

If curvature, damping, compressive mechanical forces, and nonlinear stretching effects are neglected, Equation (24) reduces to

(25) $$\begin{array}{cc} \; & \left(\rho \;A+\rho_{s}\;S_{s}\right)\frac{\partial^{2}w}{\partial t^{2}}+\frac{2\nu \;I\;\rho_{s}}{h}\frac{\partial^{4}w}{\partial x^{2}\partial t^{2}}\\ \; & +\left(EI+\left(2\mu_{s}+\lambda_{s}\right)I_{s}-\frac{2\nu \;I\;\tau_{s}}{h}\right)\frac{\partial^{4}w}{\partial x^{4}}-\tau_{s}S_{s}\frac{\partial^{2}w}{\partial x^{2}}=q\left(x,t\right) \end{array}$$

which is the same equation derived by Liu and Rajapakse [11] for the case of a straight Euler beam.

## 3. Case Studies

To test the model, the results were compared against available experimental data from literature. Two cases were considered: a nanobeam with fixed boundaries and a nano cantilever beam. In both cases, the beams were subject to static point loads.

Setting time-dependent terms in Equation (25) equal to zero and restoring initial curvature, the static deflection of a curved beam under point load is governed by

(26) $$\begin{array}{c} \left(EI-\frac{2\nu \;I\;\tau_{s}}{h}+\left(2\mu_{s}+\lambda_{s}\right)I_{s}\right)\left(\frac{\partial^{4}w}{\partial x^{4}}+\frac{d^{4}w_{o}}{dx^{4}}\right)-\tau_{s}S_{s}\left(\frac{\partial^{2}w}{\partial x^{2}}+\frac{d^{2}w_{o}}{dx^{2}}\right)=F\;\delta \left(x-a\right) \end{array}$$

where Fδ(x−a) is the Dirac–delta function representing a point load *F* applied at a distance *a* from beam support.

### 3.1. Fixed–Fixed Curved Nanobeam under Point Load

**Figure 6:**
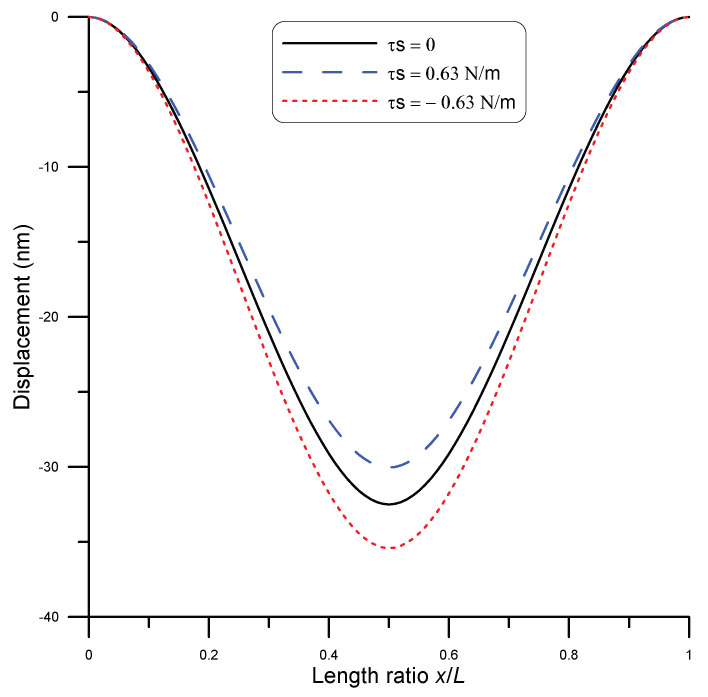
Nanobeam displacement under 62 nN point load at mid-span for the cases of $\tau_{s}=0$ (solid line), $\tau_{s}=0.63$ N/m (blue dashed line), and $\tau_{s}=-0.63$ N/m (red dotted line).

The model represented by Equation (26) can be directly integrated to study surface stress effects on the static bending of a curved nanobeam of fixed boundaries under point load. For the demonstration, a silver nanobeam of length 1 μm and 65.9 nm diameter under point load of 62 nN was used. Moreover, the beam was assumed to be initially curved with a mid-span height of 100 nm. To represent different cases of surface stress in the beam, three values of surface stress were assumed: $\tau_{s}=0.63,0,-0.63$ N/m [6]. The beam displacement is depicted in Figure 6 for each case. It was evident that a positive surface stress value increased the beam stiffness, while a negative value of surface stress resulted in a more compliant beam. These results qualitatively matched with those reported by He and Lilly [6], who also gave an explanation of the behavior in each case.

As inferred from Equation (12), the transverse stress $\sigma_{z}$ in the beam is affected by the sign of surface stress $\tau_{0}$ and beam curvature $w^{\prime \prime }$. For fixed–fixed beams, beam curvature is upward under load, which results in a negative value of $w^{\prime \prime }$. As a result, a positive value of surface stress $\tau_{0}$ produce negative transverse stress $\sigma_{z}$, which acts opposite to the direction of the applied force. This reduces the effective force on the beam. On the other hand, a negative value of $\tau_{0}$ produces a positive transverse stress $\sigma_{z}$, which, in turn, increases the effective force on the beam.

**Figure 7:**
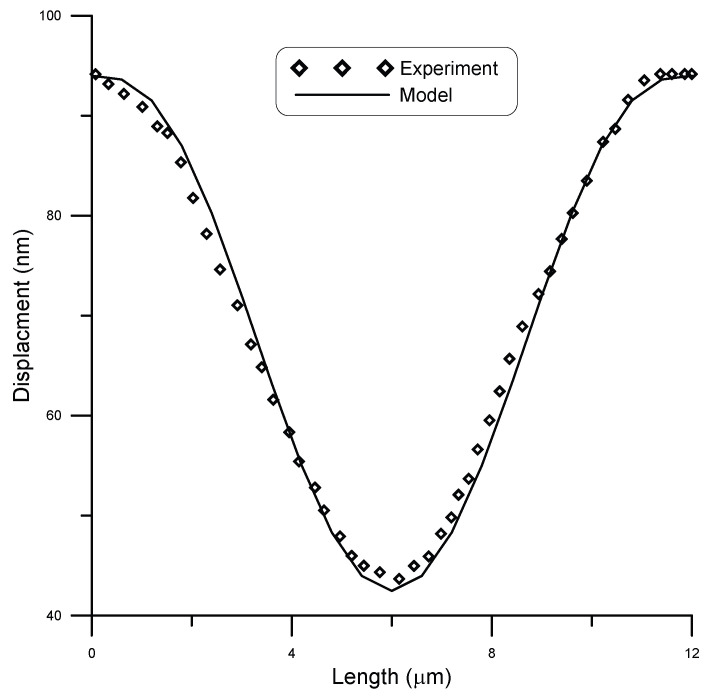
Displacement of a 12 μm long beam of 190 nm diameter under point load of 81 nN, compared to experimental data [25].

**Figure 8:**
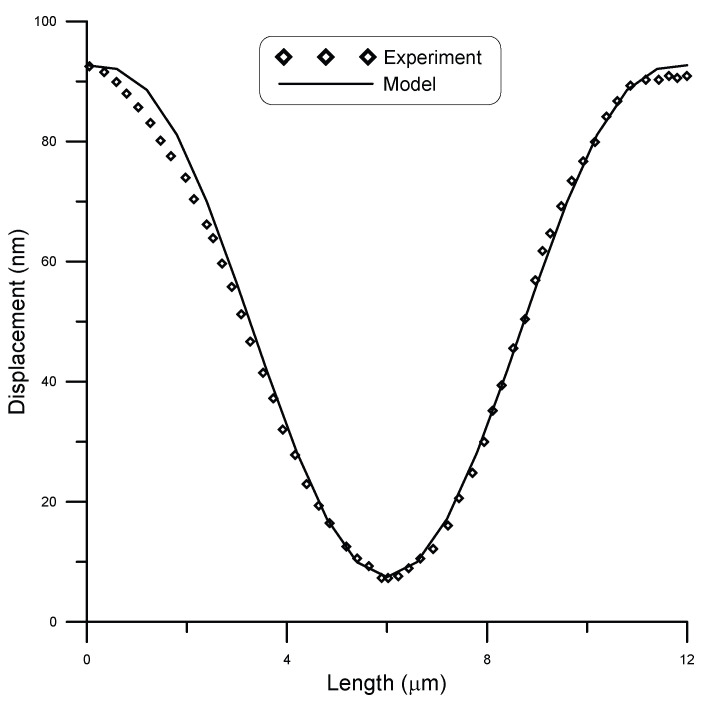
Displacement of a 12 μm long beam of 190 nm diameter under point load of 134 nN, compared to experimental data [25].

San Paulo et al. [25] experimentally tested fixed–fixed silicon nanowires of circular cross-section. They subjected initially straight wires of different cross-sections to concentrated loads and measured deflection using atomic force microscopy (AFM). A comparison between model and experimental results by San Paulo et al. [25] for the case of a 12 μm long beam and 190 nm diameter under a point load of 81 nN is given in Figure 7, while Figure 8 shows the case under a point load of 134 nN. A surface stress $\tau_{s}=0.6$ N/m [11] was assumed in the model. The figures show good agreement between model and experimental results. The coefficient of determination $R^{2}$ is a statistical measure that shows how well the model could predict the experimental outcomes. For this data set, $R^{2}\approx 0.99$ showed a strong correlation.

**Figure 9:**
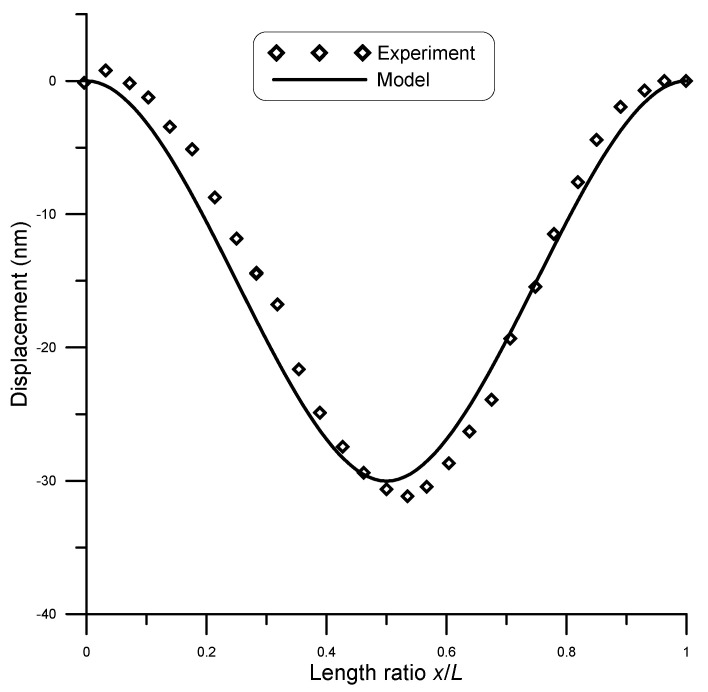
Displacement of a 1 μm long beam of 65.9 nm diameter under point load of 62 nN, compared to experimental data [26].

**Figure 10:**
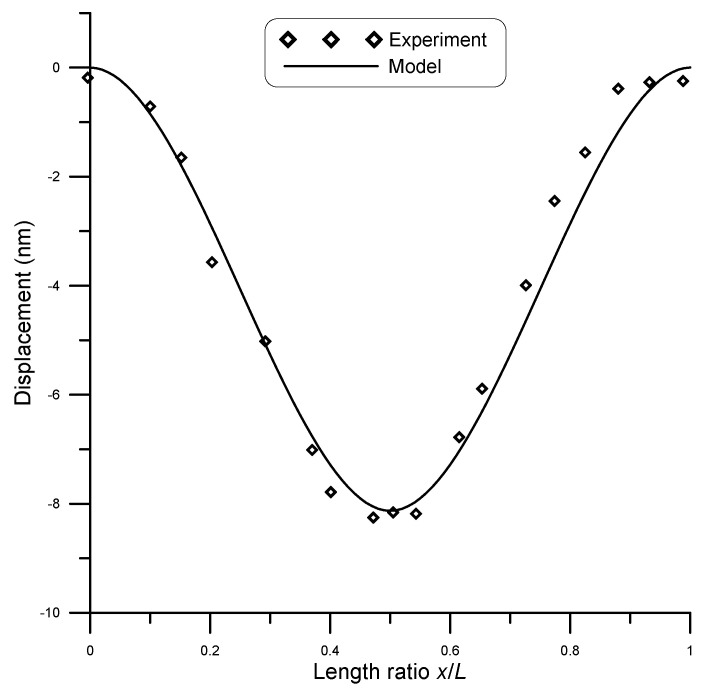
Displacement of a 1 μm long beam of 85.4 nm diameter under point load of 81.7 nN, compared to experimental data [26].

As another example, Chen et al. [26] experimentally tested fixed–fixed silver nanowires of circular cross-section. They subjected initially straight wires of different cross-sections to different point loads and measured deflection using AFM. A comparison between model and experimental results by Chen et al. [26] for the case of a 1 μm long beam with 65.9 nm diameter under a point load of 62 nN, Figure 9, and for a 1 μm long beam with 85.4 nm diameter under point load of 81.7 nN, Figure 10, was conducted. A surface stress $\tau_{s}=0.63$ N/m [6] was assumed in the model. The figures show good agreement between model and experimental results. A strong correlation between model and experimental results was observed with $R^{2}\approx 0.98$.

### 3.2. Fixed–Free Curved Nanobeam under Point Load

**Figure 11:**
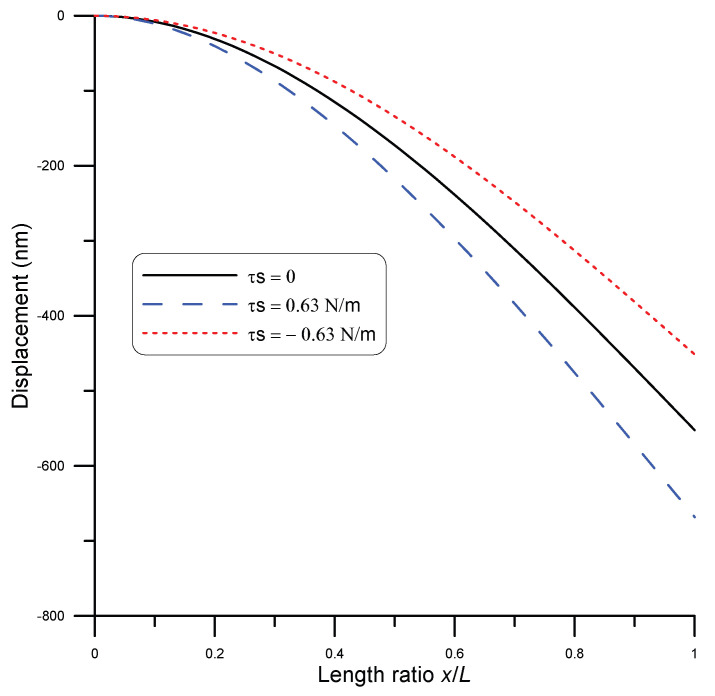
Nanobeam displacement under 1.4 nN point load at beam tip for the cases of $\tau_{s}=0$ (solid line), $\tau_{s}=0.63$ N/m (blue dashed line), and $\tau_{s}=-0.63$ N/m (red dotted line).

For cantilever nanobeams, a silver nanowire of length 2 μm, width of 150 nm, and thickness of 50 nm diameter under a point load of 1.4 nN was used [27]. Moreover, the beam was assumed to be initially curved with a tip height of 100 nm. To represent different cases of surface stress on the beam, three values of surface stress were assumed: $\tau_{s}=0.63,0,-0.63$ N/m [6]. The beam displacement is depicted in Figure 11 for each case. It was evident that a negative surface stress value increased the beam stiffness, while a positive value of surface stress resulted in a more compliant beam. These results qualitatively matched with those reported by He and Lilly [6].

As was the case for fixed–fixed beams, the transverse stress $\sigma_{z}$ in cantilever beams is affected by the sign of surface stress $\tau_{0}$ and beam curvature $w^{\prime \prime }$. For a cantilever, beam curvature is downward under load which results in a positive value of $w^{\prime \prime }$. As a result, a positive value of surface stress $\tau_{0}$ produces positive transverse stress $\sigma_{z}$, which acts in the applied force direction. This increases the effective force on the beam. On the other hand, a negative value of $\tau_{0}$ produces a negative transverse stress $\sigma_{z}$, which, in turn, decreases the effective force on the beam.

**Figure 12:**
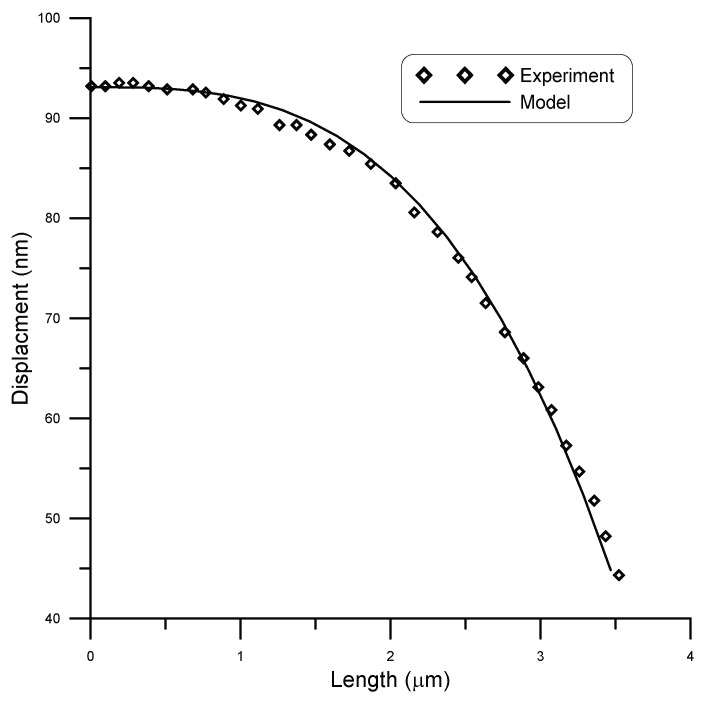
Displacement of a 3.6 μm long beam of circular cross-section of 120 nm diameter under point load of 6.6 nN, compared to experimental data [25].

**Figure 13:**
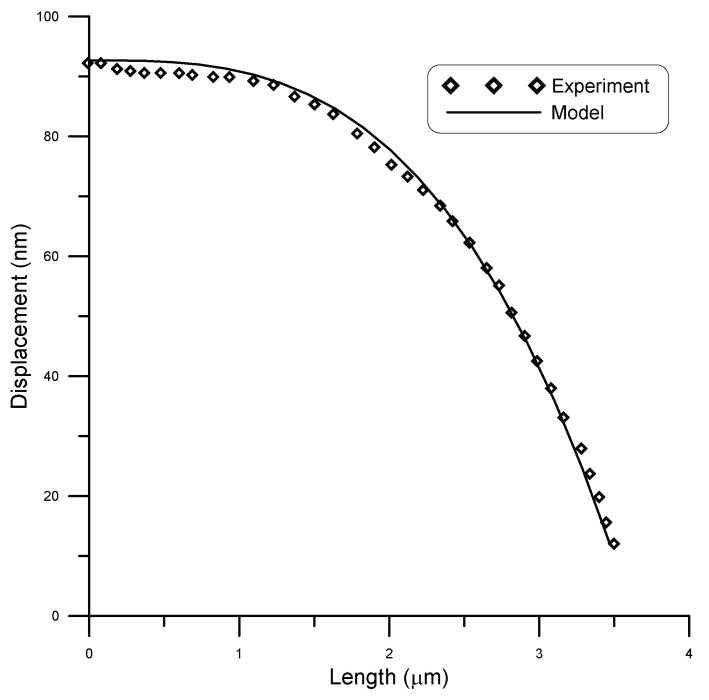
Displacement of a 3.6 μm long beam of circular cross-section of 120 nm diameter under point load of 11 nN, compared to experimental data [25].

San Paulo et al. [25] experimentally tested cantilever silicon nanobeams of circular cross-section. They fabricated nanocantilevers of 3.6 μm length and 120 nm diameter. They subjected the beam to different point loads using an AFM probe acting in contact mode with the cantilever along its length. Figure 12 shows a comparison between model and experimental results by San Paulo et al. [25] for the case of a point load of 6.6 nm, while Figure 13 shows the comparison for a point load of 11 nm. As depicted by the figures, good agreement was achieved. A strong correlation between model and experimental results was observed with $R^{2}\approx 0.99$.

Nilsson et al. [27] experimentally tested cantilever chromium nanobeams of rectangular cross-section. They fabricated nanocantilevers of 2 μm length, 150 nm width, and 50 nm thickness. They subjected the beam to different point loads using an AFM probe acting in contact mode with the cantilever along its length. At 0 N load, the cantilever was initially curved, where the tip is about 420 nm below the straight position.

**Figure 14:**
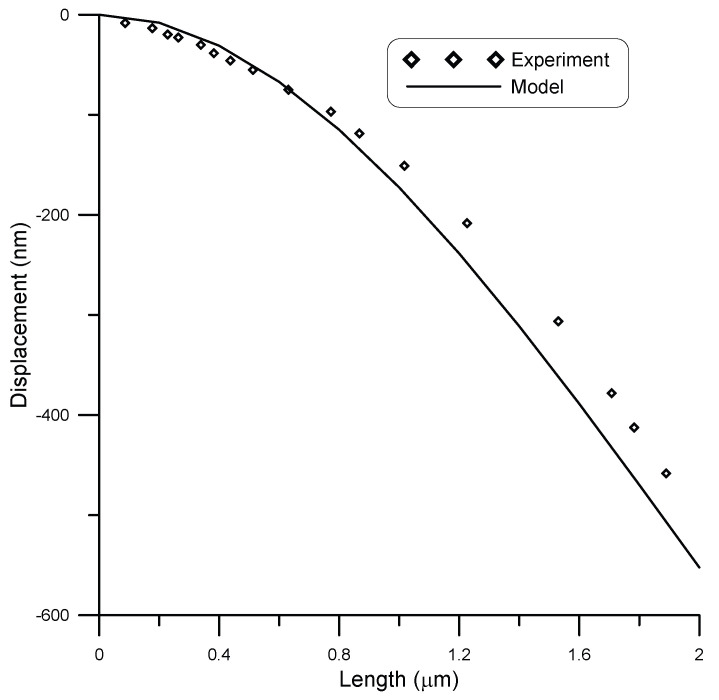
Displacement of a 2 μm long beam of rectangular cross-section of 150 nm width and 50 nm thickness under a load of 1.4 nN, compared to experimental data [27].

**Figure 15:**
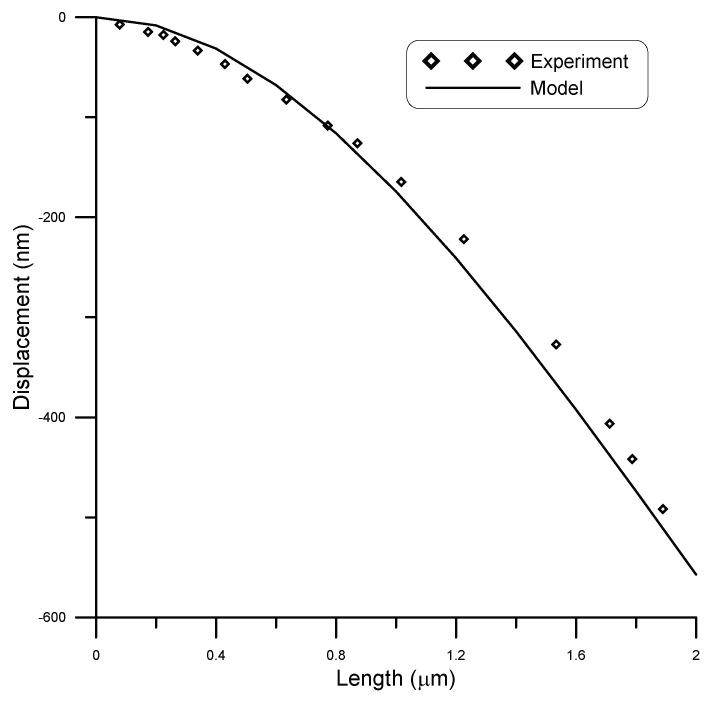
Displacement of a 2 μm long beam of rectangular cross-section of 150 nm width and 50 nm thickness under a load of 2.1 nN, compared to experimental data [27].

The initial curved shape of the cantilever was considered in the model, and the results were compared against experiments, as seen in Figure 14 and Figure 15. Figure 14 shows the displacement of the cantilever beam under a point load of 1.4 nN, while Figure 15 shows the displacement of the cantilever beam under a point load of 2.1 nN. Good agreement was obtained between model and experimental results with $R^{2}\approx 0.97$. The reason for the slight mismatch between model and experimental results is the fact that the model results were calculated assuming no surface stress in the beam, due to lack of information of surface stress parameters for chromium nanowires.

## 4. Conclusions

In this work, a model, describing nanowires and accounting for curvature and surface stress, produced. A static version of the model is used to study the static bending of fixed–fixed and fixed–free nano beams under point loads. The model was verified by contrasting its findings with experimental data that was published in the literature for both types of beams, achieving good agreements.

The results showed that a positive surface stress value increased the stiffness of fixed–fixed beams, while it resulted in a decrease in stiffness for fixed–free beams. The case was reversed if surface stress was negative.

Finally, the model results indicated the viability of the present model for curved nanobeams, incorporating surface energy effects. However, further work needs to be conducted to test the model for various applied loading conditions.

## Data Availability

Not applicable.
